# Multiple Benefits of Empagliflozin in PCOS: Evidence from a Preclinical Rat Model

**DOI:** 10.3390/pathophysiology31040041

**Published:** 2024-10-09

**Authors:** Dejana Rakic, Vladimir Jakovljevic, Vladimir Zivkovic, Jovana Jakovljevic Uzelac, Nikola Jovic, Maja Muric, Bozidar Pindovic, Aleksandra Dimitrijevic, Petar Arsenijevic, Jovan Rakic, Slobodanka Mitrovic, Tatjana Vulovic, Jovana Joksimovic Jovic

**Affiliations:** 1 Department of Gynecology and Obstetrics, Faculty of Medical Sciences, University of Kragujevac, 34000 Kragujevac, Serbia; dejavulovic@gmail.com (D.R.); docctorny@gmail.com (N.J.); saskadkg@gmail.com (A.D.); petar.arsenijevic@yahoo.com (P.A.); 2 University Clinical Center Kragujevac, Zmaj Jovina 30, 34000 Kragujevac, Serbia; majanikolickg90@gmail.com (M.M.); smitrovic@medf.kg.ac.rs (S.M.); tatjana_vulovic@yahoo.com (T.V.); 3 Department of Physiology, Faculty of Medical Sciences, University of Kragujevac, 34000 Kragujevac, Serbia; drvladakgbg@yahoo.com (V.J.); vladimirziv@gmail.com (V.Z.); pindovic.bozidar@gmail.com (B.P.); 4 Center of Excellence for Redox Balance Research in Cardiovascular and Metabolic Disorders, 34000 Kragujevac, Serbia; 5 Department of Human Pathology, I.M. Sechenov First Moscow State Medical University, 119146 Moscow, Russia; 6 Department of Pharmacology, I.M. Sechenov First Moscow State Medical University, 119435 Moscow, Russia; 7 Institute of Medical Physiology “Richard Burian”, Faculty of Medicine, University of Belgrade, 11000 Belgrade, Serbia; jovanavjakovljevic@gmail.com; 8 Department of Pharmacy, Faculty of Medical Sciences, University of Kragujevac, 34000 Kragujevac, Serbia; 9 Department of Dentistry, Faculty of Medical Sciences, University of Kragujevac, 34000 Kragujevac, Serbia; rakic999@hotmail.com; 10 Department of Pathology, Faculty of Medical Sciences, University of Kragujevac, 34000 Kragujevac, Serbia; 11 Department of Surgery, Faculty of Medical Sciences, University of Kragujevac, 34000 Kragujevac, Serbia

**Keywords:** polycystic ovary syndrome, metformin, empagliflozin, rats, sodium-glucose cotransporter type 2 inhibitors, metabolic syndrome, reproduction

## Abstract

Polycystic ovary syndrome (PCOS) is the most common complex endocrinological condition of women that is associated with infertility and metabolic disorders during the reproductive period. Recently, a great deal of research has focused on the etiopathogenesis of this disorder and the modulation of therapeutic approaches. There are still many controversies in the choice of therapy, and metformin is one of the most commonly used agents in the treatment of PCOS. Considering the link between metabolic disorders and PCOS, glycemic status is crucial in these patients, and sodium-glucose cotransporter type 2 inhibitors (SGLT2is) represent a potentially promising new therapeutic approach. These drugs have been shown to improve glucose metabolism, reduce adipose tissue, decrease oxidative stress, and protect the cardiovascular system. These data prompted us to investigate the effects of empagliflozin (EMPA) in a PCOS rat model and compare them with the effects of metformin. We confirmed that EMPA positively affects somatometric parameters, glucose and lipid metabolism, and the levels of sex hormones, as well as reduces oxidative stress and improves ovarian function and morphology. Administration of EMPA at doses of 5 mg/kg, 15 mg/kg, and 45 mg/kg during a 4-week treatment period improved, as induced by estradiol valerate and a high-fat diet, the metabolic and reproductive statuses in a PCOS rat model. The best effects, which were comparable to the effects of metformin, were achieved in groups receiving the middle and highest applied doses of EMPA. These results may prompt further clinical research on the use of EMPA in patients with PCOS.

## 1. Introduction

Polycystic ovary syndrome—PCOS—is the most common complex endocrinological condition of women during the reproductive period [1]. Often this endocrinopathy is associated with infertility, abdominal obesity, insulin resistance, acne, hirsutism, and other metabolic disorders [1]. PCOS is considered a complex polygenetic and multifactorial disorder with a strong epigenetic impact [2]. Moreover, environmental factors also have an important role in the development of PCOS; recent research has proven that increased concentrations of phthalates may be in correlation with reproductive and metabolic features in PCOS women [3]. During the last few decades, numerous conferences have discussed which diagnostic criteria are relevant for establishing a diagnosis. Twenty years ago at an expert conference in Rotterdam, it was decided that, in order to establish a diagnosis, the presence of two of three criteria is necessary: (I) oligo-amenorrhea, (II) hyperandrogenism (clinical or biochemical), and (III) the presence of polycystic ovary morphology confirmed by ultrasound (PCOS ovary phenotype—O-PCOS) [4]. O-PCOS is described as an ovarian volume greater than 10 mL^3^ and/or more than 20 follicles present on at least one ovary [4]. Today, in order to diagnose PCOS, the Rotterdam consensus is applied in all relevant institutions. Two PCOS phenotypes are frequently presented: (I phenotype) women who have ovulatory menstrual cycles and hyperandrogenism, and (II phenotype) women who have oligoovulatory or anovulatory cycles without the presence of increased androgen secretion and with the presence of polycystic ovarian morphology confirmed by ultrasound [5]. Reproductive abnormalities are considered to be due to inadequate gonadotropin stimulation, hyperandrogenism, chronic anovulation, and polycystic ovarian morphology. The most common disorder in the secretion of gonadotropin is a changed mean value of LH due to an increase in the frequency and amplitude of its secretion [6,7]. Serum FSH concentrations in PCOS patients are normal or decreased [8]. Preantral, small follicles due to insufficient FSH stimulation increase androgen synthesis and lead to hyperandrogenism, which is a key pathophysiological disorder in PCOS [9]. There are studies that also claim that oxidative stress (OS) is associated with adverse pregnancy outcomes in PCOS patients [10]. Previous review papers have focused on OS imbalance and oocyte development and the association between hyperandrogenism and OS, but there are newer studies that have also investigated the impact of OS on the endometrium. Research has shown that endometrial receptors are reduced in PCOS and that OS also occurs in the endometrium of PCOS subjects [11]. Inflammatory mediators cause damage to endometrial receptors, and they disrupt endometrial decidualization and embryo implantation [12]. The proof that OS forms an integral part of the pathogenesis of PCOS is that, due to the use of supplements in PCOS therapy, the use of antioxidants has a positive effect [13]. Therapeutic and curative treatment should regulate hyperandrogenism and the menstrual cycle, induce ovulation, and regulate metabolic disorders (such as hyperinsulinism and dyslipidemia) to prevent endometrial hyperplasia and decrease the BMI in those with the obese PCOS phenotype. There are numerous therapeutic approaches, such as the initially introduced hygienic-dietary regime, and the application of metformin (MET), which is considered the gold standard of therapy thus far [14]. Despite extensive efforts to discover effective treatments for PCOS, a definitive protocol has not been proposed, and therapy continues to be primarily symptomatic.

Selective SGLT-2i are proteins, and they are also members of the SGLT family of sodium and glucose transporters. The effect of SGLT2i does not depend on insulin secretion from pancreatic beta cells or insulin resistance [15,16]. They participate in the secondary active transport of glucose and sodium molecules according to the concentration gradient. SGLT2 is a high-capacity, low-affinity transporter responsible for the reabsorption of 90% of the glucose in the proximal tubule of the kidney [17]. Glucose transport by SGLT2 is carried out by symporter, and sodium and glucose pass through the tubulocyte membrane in the same direction according to the concentration gradient [18]. The energy stored in the electrochemical gradient of sodium enables the movement of glucose toward its gradient [17]. The inhibition of SGLT2 reduces the glucose level by preventing the reabsorption of glucose in the proximal tubule, which leads to the excretion of glucose through urine. Glycosuria reduces the glucose level by a third of its value [19,20]. Many studies have shown that SGLT2i reduces the amount of adipose tissue, lowers arterial blood pressure, reduces oxidative stress, and possesses anti-inflammatory and cardioprotective properties [17]. A loss of body mass predominantly results in a loss of adipose tissue [21]. The current therapeutic application has proven to induce a positive effect in the regulation of glycemic status disorders and cardiovascular function, and it additionally increases a possible successful effect in the treatment of PCOS [22,23] The use of SGLT2i has been proven to be promissive in the treatment of hormonal and metabolic issues, as well as for somatometric parameters of PCOS [23]. The results thus far have shown that the use of SGLT2i in women with PCOS leads to a regulation of the body weight and lipid metabolism, to a secretion of insulin and androgens, and to a decrease in blood pressure [24]. It has been proven that they can lead to weight loss in women who do not suffer from diabetes mellitus type 2 [25,26,27]. Javed et al. [27] concluded that SGLT2i can improve anthropometric parameters, and Sinha et al. [28] concluded that SGLT2i can produce beneficial outcomes on the metabolic status and hormonal effect in PCOS patients. Only a few studies on the impact of SGLT2i on PCOS rat models exist. Pruett [29] studied oxidative stress in PCOS rat adipose tissue. The mechanism by which SGLT2 acts on the pathophysiological changes in PCOS is the reduction in oxidative stress, which is mediated by the exchange of sodium hydroxide and the inhibition of nicotinamide dinucleotide phosphate [30]. However, there are no mechanistic studies that have examined the effects of SGLT2i on the reproductive and metabolic parameters in a PCOS rat model. Although SGLT2i has not yet been registered in the treatment of PCOS [31], the 2023 International Guideline for the Treatment of PCOS emphasizes the importance of weight regulation and suggests that drugs that lead to weight loss could be very useful in the treatment of obese PCOS patients [32]. Thus far, no drug has been found that would act on this entire spectrum of abnormalities. Despite progression in the revealing of the unknown characteristics of PCOS, the therapeutic approaches that persist are incomplete and insufficient. These findings, as well as the doubts related to the exact etiopathogenesis and treatment of PCOS, have led us to investigate the potential benefits of using SGLT2i in an experimentally induced PCOS model. This study addresses a significant gap in the current understanding of SGLT2i’s effects on PCOS. While previous research has shown promising results for SGLT2i in managing some aspects of PCOS, there is a lack of comprehensive mechanistic studies examining their impact on both reproductive and metabolic parameters in a PCOS rat model. This research aims to provide a more comprehensive understanding of SGLT2i’s potential in PCOS treatment, which could lead to new therapeutic strategies. The primary aim of this study is to investigate the effects of SGLT2i on reproductive and metabolic parameters in an experimentally induced PCOS rat model. By examining these effects, we seek to elucidate the potential mechanisms by which SGLT2i might ameliorate the complex symptoms associated with PCOS, including hormonal imbalances, metabolic disorders, and oxidative stress. This research aims to provide valuable insights that could inform future clinical applications of SGLT2i in PCOS treatment and contribute to the development of more targeted and effective therapeutic approaches for this complex endocrine disorder.

## 2. Materials and Methods

### 2.1. Ethics Statement

This investigation was conducted at the Faculty of Medical Sciences, University of Kragujevac (Kragujevac, Serbia), and the Faculty’s Ethical Committee for experimental animal well-being approved ethical permission (protocol code 01-10636, dated 4 October 2022). All protocols were performed in accordance with the European Council Directive (No. 86/609/EEC), Good Laboratory Practice principles, and ARRIVE guidelines. Every attempt was made to minimize the number of animals used and sacrificed.

### 2.2. Animals

The research was conducted on 42 rats (control group—6 animals and PCOS group—36 animals) that were of the Wistar albino strain, female, 6 weeks old, and had an average body weight (150–170 g). The animals were purchased from the Department of Laboratory and Experimental Animal Breeding, Military Medical Academy, Belgrade. The animals were housed (3 animals per cage) in controlled environmental conditions (temperature—23 ± 1 °C, light/dark cycle—12/12 h) with unlimited access to food and water during the duration of the protocol (ad libitum). All animals underwent estrus cycle examination, and only those with regular (4–5 days) cycling were enrolled in the experimental protocol.

### 2.3. Experimental Protocol

#### 2.3.1. Assessment of the Estrus Cycle

In order to detect the phase of the estrus cycle, vaginal smears and cytological examinations were performed. Using a dropper filled with small volume of saline, vaginal lavage was conducted, and the samples were placed on a glass slide, stained with hematoxylin, and analyzed by a light microscope. The phases of the estrus cycle are as follows: proestrus (characteristic—round nucleated cells), estrus (characteristic—cornified squamous cells), metaestrus (characteristic—cornified squamous cells mixed with leucocytes), and diestrus (characteristic—nucleated epithelial cells and a predominance of leucocytes) [33]. Detection of the phases of the estrus cycle was performed in 12 days.

#### 2.3.2. PCOS Induction

Rats with a regular estrus cycle were randomly divided into 2 groups. The first group (control group, *n* = 6) subcutaneously received olive oil (as a solvent and in the same volume as the PCOS group) once. Another group (PCOS group, *n* = 36) received estradiol valerate (4 mg in 0.4 mL of olive oil) along with a high-fat diet regime [34,35]. The combination of the hormone estradiol valerate and fatty food caused reproductive and metabolic changes that simulated the obese PCOS phenotype, as we have previously demonstrated [23]. To confirm PCOS, 6 animals from the control group, as well as 6 animals from the PCOS group, were sacrificed by decapitation on the guillotine after anesthesia (50 mg/kg of ketamine and 100 mg/kg of xylazine). After confirmation of successful PCOS induction, the rats from the PCOS group were further divided into 5 groups (6 animals per group) and treated as follows: distilled water, EMPA at a dose of 5 mg/kg BW, EMPA at a dose of 15 mg/kg BW, EMPA in dose of 45 mg/kg body weight [29], and MET in a dose of 500 mg/kg body weight [36]. The mentioned treatments were carried out by gavage, every day, during the 28 days of the treatment. Doses of the estradiol valerate [34], EMPA [29], and MET [36] were chosen based on data from the literature. This study employed a comprehensive dosing strategy utilizing three escalating doses of EMPA to investigate potential dose-dependent effects, while also including MET as a positive control to benchmark the SGLT2i’s efficacy against the current gold standard treatment for PCOS. 

Twenty-eight days after induction of PCOS, the animals were anesthetized (50 mg/kg of ketamine and 100 mg/kg of xylazine) and sacrificed by decapitation on a guillotine, and blood and tissue from the animals were then sampled for further analysis.

### 2.4. Somatometric Parameters 

The body weight of the animals was measured at the beginning of the protocol after induction of PCOS, as well as twice a week during treatment with EMPA and MET. During the treatment, it was necessary to determine the body weight in order to recalculate the dose of EMPA and MET that was needed for administration to the animals. The body weight was measured when cleaning the cage, and then the dose that the rats would receive for the next 3–4 days was recalculated. The calculated dose of the applied drug was increased in accordance with the increase in the body weight of the animals. After the treatment with EMPA and MET, as well as immediately before sacrifice, the animals were weighed. Immediately before sacrifice, the body mass was measured, which represented the final body mass, and the body mass yield was calculated as a percentage increase in relation to the initial body mass.

### 2.5. Blood Pressure Assessment

Blood pressure was measured by tail plethysmography at the end of the experimental protocol. A non-invasive blood pressure measurement method using the BP system method (Rat Tail Cuff Method Blood Pressure Systems (MRBP-R), IITC Life Science Inc. Woodland Hills, CA, USA) allowed for an accurate measurement of the systolic, special diastolic, mean pressure, and air pressure values to detect the changes in the volume internal blood vessel pressure (VPR sensor).

### 2.6. Oral Glucose Tolerance Test (OGTT) 

At 48 h before sacrifice, all of the animals were subjected to fasting for 12 h, and then they were given a glucose gavage at a dose of 2 g/kg. Blood samples were taken at 30, 60, and 120 min after glucose administration. The glucose level was measured with a glucometer (Accu-Chek, Roche 165 Diagnostics, Indianapolis, IN, USA) with appropriate strips.

### 2.7. Biochemical Analysis

#### 2.7.1. Collection of the Blood and Tissue Samples for Biochemical and Histological Analyses

In order to obtain the plasma/serum, blood was collected in tubes after executive decapitation was performed. Then, the centrifuge was performed at 3000 RPM, and the sampled plasma/serum and erythrocyte lysates were stored at −20 °C until time for the analysis.

#### 2.7.2. Hormonal Assays

The serum samples were used to detect the levels of the following sex hormones: total testosterone (T), progesterone (P), and estradiol (E2). The electrochemilumnescent immunoassay (ECLIA) method was used with an Elecsis 2010 analyzer in order to detect the levels of T, P, and E2. Standard commercial kits (Elecsis Testosterone II, Progesterone II, and Estradiol III Roche Diagnostics, Mannheim, Germany) were applied. The levels of T and P were expressed in ng/m L, while E2 was expressed in pg/m L. The sensitivity of the T, P, and E2 was 0.025 ng/mL, 0.03 ng/mL, and 5 pg/mL, respectively. Commercial kits (Elabscience^®^, Houston, TX, 77079, USA) were used for the detection of the LH and FSH concentrations of the mentioned hormones, which were then determined by the ELISA method. The following reagents were used for the analysis: Rat LH (luteinizing hormone) ELISA Kit, catalog number—E-EL-R0026; Rat FSH (follicle stimulating hormone) ELISA Kit, catalog number—E-EL-R0391. The samples (as well as the standards) were added to the wells of the micro ELISA plate and mixed with the specific antibody. Then, a biotin detection antibody specific for the appropriate rat hormone and conjugated Avidin-Horseradish peroxidase (HRP) were added sequentially to each well. After the incubation, the microplates were washed in accordance with the instructions. A substrate solution was added to each well, and only those wells containing the primary antibody specific for the corresponding hormone were stained blue. The enzyme–substrate reaction was stopped by adding a stop solution, after which the color turned yellow. The optical density (OD) was spectrophotometrically determined at a wavelength of 450 nm ± 2 nm. The OD value was corresponding to the hormone concentration. The hormone concentration was calculated in the samples by comparing the samples with a standard curve. The coefficients of variation for each of the hormones were less than 10%.

#### 2.7.3. OS Parameters in the Blood Samples

From the plasma and erythrocyte lysates, the values of the parameters of the antioxidant protection system, as well as the pro-oxidants, were determined: the lipid peroxidation index was measured as TBARS; and the nitrogen monoxide was measured in the form of nitrite (NO_2_^−^), superoxide anion radical (O_2_^−^), hydrogen peroxide (H_2_O_2_), catalase (CAT), superoxide dismutase (SOD), and reduced glutathione (GSH).

##### TBARS Determination

Calculation of TBARS was conducted after of 0.8 mL of plasma sample and 0.4 mL of trichloroacetic acid were mixed. After cooling the mixture, it was stored on ice for 15 min, and then the supernatant was sampled. Using 1% thiobarbituric acid in 0.05 NaOH, incubation was performed with the sampled supernatant at 100 °C for 15 min. It was then measured at a 530 nm wavelength. As a blank probe, the solution of distilled water was used [37,38].

##### NO_2_^−^ Determination

The index of the NO production represents the NO_2_ level, and its determination was performed using Griess reagent. In total, 0.1 mL of 3 N perchloric acid, 0.4 mL of 20 mM ethylenediaminetetraacetic acid, and 0.2 mL of the sample was stored on ice for 15 min and then centrifuged for 15 min at 6000 rpm. After pouring off the supernatant, 220 μL of K_2_CO_3_ was added. The concentration of NO_2_ was measured at a wavelength of 550 nm, and distilled water was used as a blank probe [39].

##### O_2_^−^ Determination

After completion of the nitro reaction of tetrazolium blue with TRIS buffered with the plasma samples, the concentration of superoxide anion radical (O_2_^−^) was determined and measured. The concentration was measured at a wavelength of 530 nm. Distilled water was used as a blank probe [40].

##### H_2_O_2_ Determination

The concentration of hydrogen peroxide (H_2_O_2_) was determined based on the principle of oxidation of the phenol red hydrogen peroxide in a reaction catalyzed by horseradish peroxidase. A total of 200 μL of plasma sample was precipitated with 800 μL of freshly prepared phenol red solution, after which 10 μL (1:20) of HRP was added. The H_2_O_2_ level was measured at a wavelength of 610 nm, and distilled water was used as a blank probe [41].

##### CAT Determination

In order to measure the CAT activity, a total of 50 μL of CAT buffer, 100 μL of sample, and 1 mL of 10 mM H_2_O_2_ were used. Detection was performed at a wavelength of 360 nm. Distilled water served as a blank probe, and the amount of CAT was expressed as U/g hemoglobin × 103 [42].

##### Determination of SOD

The SOD activity was determined by epinephrine. A total of 100 μL of sample and 1 mL of carbonate buffer were mixed, and then 100 μL of epinephrine was added. Detection was performed at a wavelength of 470 nm, and the amount of SOD was expressed as U/g hemoglobin × 103 [43].

##### Determination of GSH

The oxidation of reduced glutathione (GSH) was used to measure the level of GSH via 5,5-dithiobis-6,2-nitrobenzoic acid. The GSH extract was made by combining 0.1 mL of 0.1% EDTA, 400 μL of plasma, and 750 μL of precipitation solution (containing 1.67 g of metaphosphoric acid, 0.2 g of EDTA, and 30 g of NaCl, which were made up to 100 mL with water). Mixing and cooling on ice was performed in a vortex machine for 15 min. The measurement was made at a wavelength of 420 nm, while distilled water was used as a blank probe [44].

### 2.8. Ovarian and Adipose Tissue Histology

The left ovary, periovarian, and subcutaneous adipose tissue were isolated and fixed in 4% formaldehyde at room temperature. After fixation, the tissue samples were dehydrated through a series of alcohols of increasing concentration (50, 70, 96, and 100%) and were illuminated in xylene and molded in paraplast. Transverse serial sections of ovarian tissue, 5 μm thick, were cut on a rotary microtome. After drying, the tissue sections were deparaffinized in xylene and then rehydrated in decreasing concentrations of alcohol (100, 96, 70, and 50%), which were washed in water and then stained with Mayer’s hematoxylin and 2% eosin solution. After staining, sections were mounted with DPX and coverslipped. Morphometric analysis of the ovaries was carried out by analyzing the sections stained with hematoxylin and eosin. Quantification of the primary, secondary, tertiary, atretic, and cystic follicles, as the number of corpora lutea, was performed on sections of ovarian tissue using a light microscope (Olympus BX51). For follicle quantification, 3 sections per ovary were selected: the central section, and 150 μm in front and 150 μm behind the central section [45]. Only those follicles that had a visualized oocyte and nucleus were counted. The classification of follicles was as follows (according to established methodology): preantral, (healthy) antral, and atretic follicles [45]. The corpora lutea and the number of cystic follicles were counted from the central section. The characteristics for cystic change in the follicle were as follows: single-layered-to-four-layered granulosa cells, without luteinization, thin wall made of thin flattened cells on a thin fibrous capsule, and enlarged dimensions. The adipocyte diameter and adipocyte surface area were measured using the Adiposoft plugin for ImageJ (1.53e).

## 3. Results

### 3.1. Impact of the EMPA and MET on the Somatometric Indices, Estrus Cycle, Glycoregulation, and Blood Pressure

**Figure 1:**
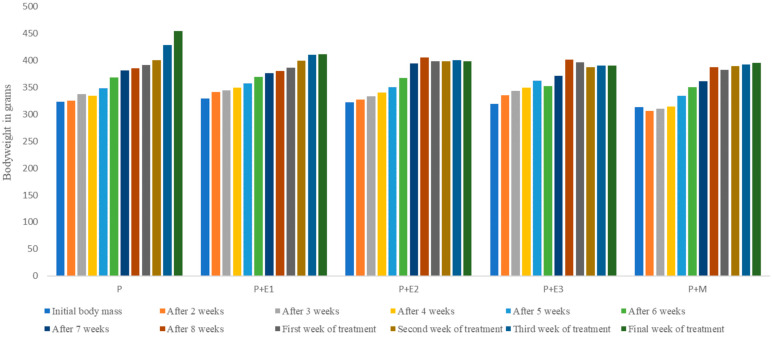
Bodyweight changes during the duration of the experiment.

**Figure 2:**
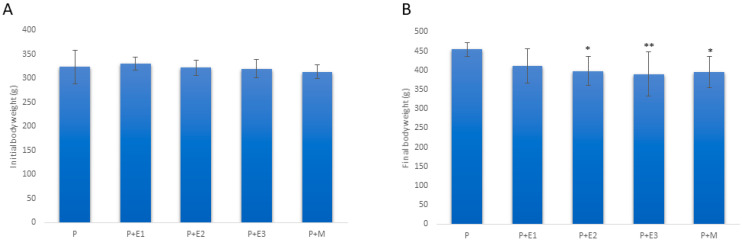
Body weight changes before (**A**) and after (**B**) EMPA and MET treatments. * statistical significance at level *p* < 0.05 compared to the P group; ** statistical significance at level *p* < 0.01 compared to the P group.

Throughout the experimental protocol, no statistically significant differences in body mass were observed among the individual groups. Figure 1 illustrates the body weight trends of the rats, indicating a general increase over the course of the study. Group P exhibited the highest increase in body weight, while other treatment groups also showed a positive trend in body weight gain, though they were not statistically significant. Figure 2 demonstrated that initial body weight were not statistically different between groups (Figure 2A), but the final body weight was significantly lower after middle and the highest EMPA dose treatment as well as after MET treatment (Figure 2B).

**Figure 3:**
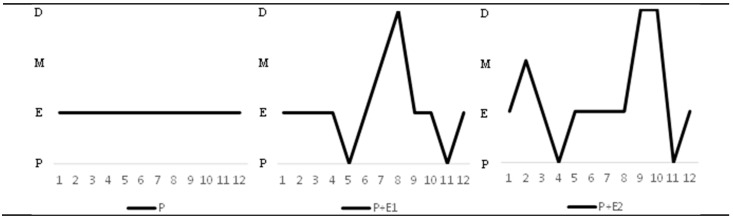

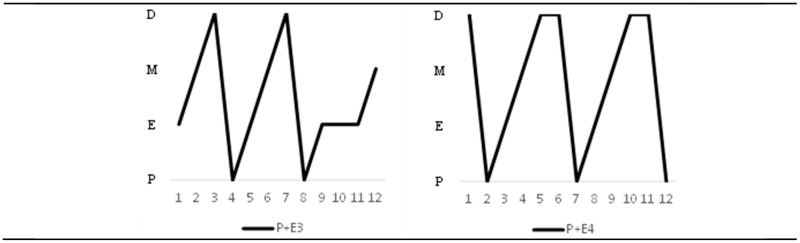
Changes in the estrus cycle in five rats induced with PCOS and after the applied treatments. Y-axis legend: D—diestrus, M—metaestrus, E—estrus, and P—proestrus.

Figure 3 demonstrates the representative animals from each group. All animals from the P group were in the estrous phase after PCOS induction and remained in persistent estrus for the last 12 days, whereas those treated with EMPA and MET showed a tendency to establish a regular estrus cycle.

**Figure 4:**
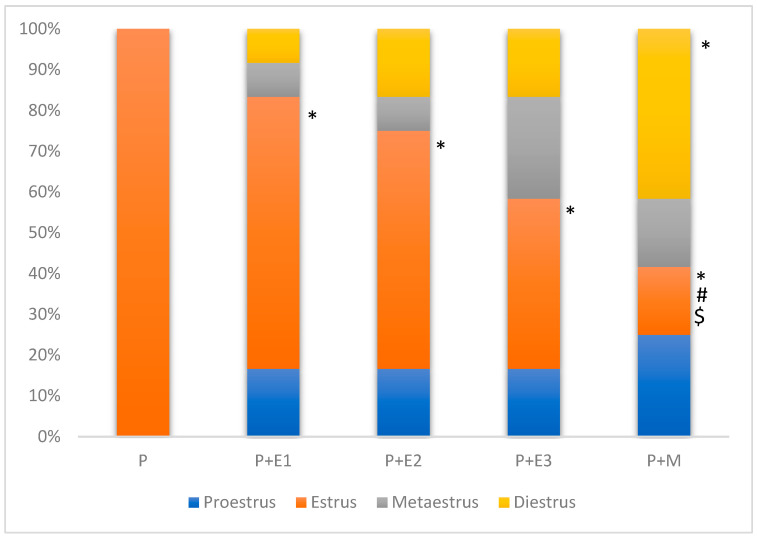
The proportions of different estrus cycle stages in the three groups were analyzed using the Chi-square test, and the differences were evaluated by z-test. * statistically significant difference compared to the P group (*p* < 0.05), # statistically significant difference compared to the P+E1 group (*p* < 0.05), and $ statistically significant difference compared to the P+E2 group (*p* < 0.05).

Moreover, as shown in Figure 4, the rats from P group were constantly in the estrus phase during examination, while all of the treated groups showed different proportions of other estrus cycle phases. All of the treatments were significantly reduced in their percentage of the estrus phase, while the MET-treated group were significantly reduced in their percentage of estrus compared to all of the EMPA-treated groups.

As shown in Table 1, the basal values of glycemia were statistically significantly different between the examined groups in relation to the P group (*p* < 0.01). The glycemia values at 30 min were also statistically significantly different in relation to the P group (*p* < 0.01). At 30 min, a statistically significantly lower value of glycemia was shown in the P+E3 group compared to P+E1, P+E2, and P+M groups (at *p* < 0.05). The measured glycemia values of the examined P+E1, P+E2, P+E3, and P+M groups at 60 min were statistically significantly different compared to the P group (*p* < 0.01). In addition, a statistically significantly lower value of glycemia was shown in the P+E3 group compared to the P+E1 group (*p* < 0.05). After 120 min, a statistically significant difference in the glycemia of the examined P+E1, P+E2, P+E3, and P+M groups was shown compared to the P group (*p* < 0.01).

**Figure 5:**
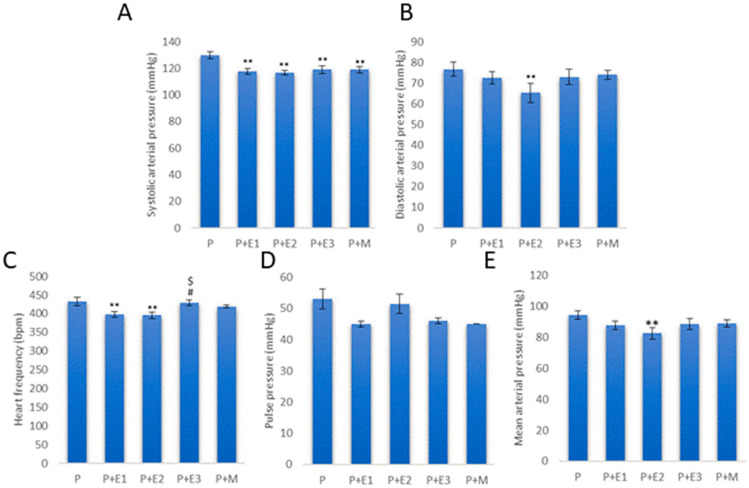
The blood pressure parameters in different groups. (**A**) systolic arterial pressure; (**B**) diastolic arterial pressure; (**C**) heart frequency; (**D**) pulse pressure; and (**E**) mean arterial pressure. Bars represent the means ± SEM. ** statistical significance at level *p* < 0.01 compared to the P group; # statistical significance at level *p* < 0.05 compared to the P+E1 group; and $ statistical significance at level *p* < 0.05 compared to the P+E2 group.

At the end of the experimental protocol, the blood pressure was measured via tail plethysmography. All of the groups treated with EMPA, as well as the group treated with MET, exhibited statistically significantly lower systolic blood pressure values compared to the P group (*p* < 0.01). Only the administration of the middle dose of EMPA resulted in a significant reduction in diastolic blood pressure, along with a decrease in the mean arterial pressure, compared to the P group (*p* < 0.01). The heart rate was lower in the groups treated with the lowest and middle doses of EMPA compared to the P group (*p* < 0.01). The pulse pressure did not significantly differ between the observed groups (*p* > 0.05), as shown in Figure 5.

### 3.2. Impact of the EMPA and Met on the PCOS-Related Alterations of the Systemic Oxidative Status and Sex Hormone Levels

**Figure 6:**
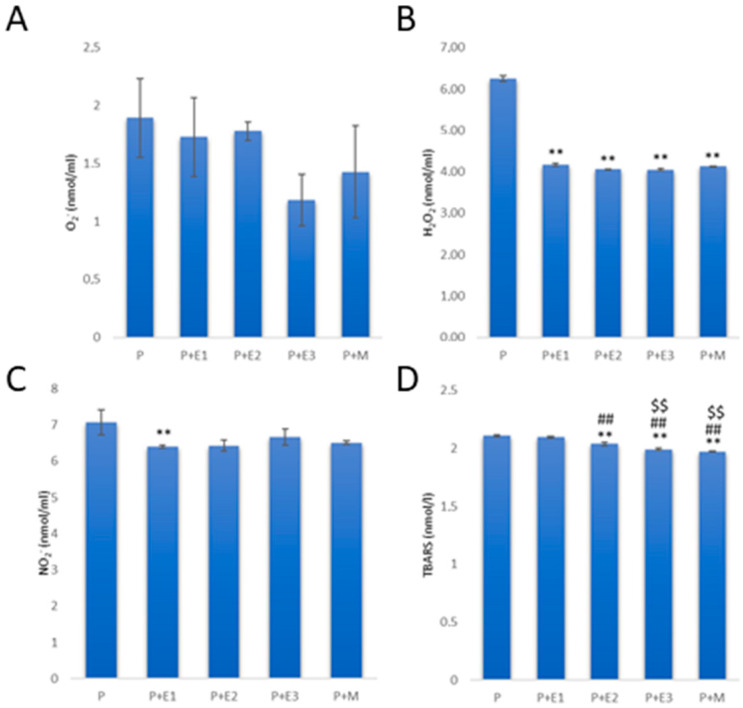
Markers of oxidative stress in different groups. (**A**) levels of O_2_^−^; (**B**) levels of H_2_O_2_; (**C**) levels of NO_2_; (**D**) levels of TBARS. Bars represents the means ± SEM. ** statistical significance at level *p* < 0.01 compared to the P group; ## statistical significance at level *p* < 0.01 compared to the P+E1 group; and $$ statistical significance at level *p* < 0.01 compared to the P+E2 group.

Pro-oxidative parameters, namely H_2_O_2_ and TBARS, were significantly higher in the control group compared to all of the treatment groups (*p* < 0.01). All doses of EMPA (EMPA) and MET lowered H_2_O_2_ levels, while the middle and highest doses of EMPA and Met decreased TBARS levels. Nitrite (NO_2_^−^) levels were only lower in the P+E1 group compared to the control group (*p* < 0.01) (Figure 6).

**Figure 7:**
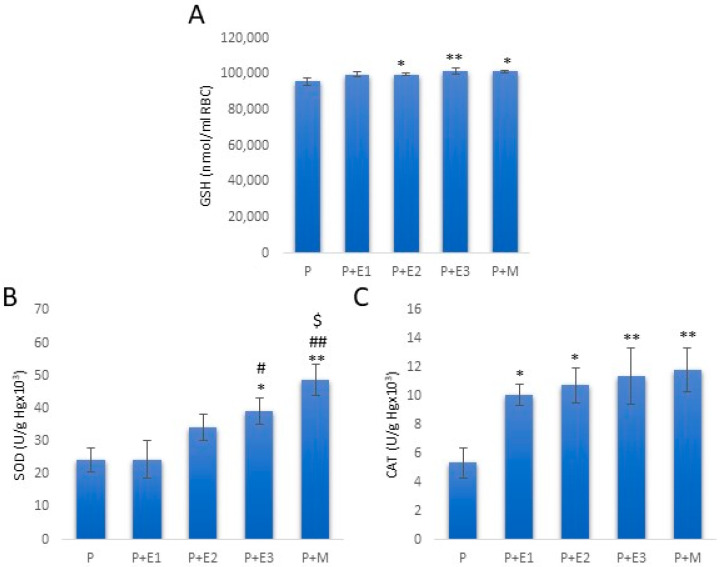
The levels of anti-oxidative enzymes in different groups. (**A**) levels of GSH; (**B**) levels of CAT; and (**C**) levels of SOD. Bars represent the means ± SEM. * statistical significance at level *p* < 0.05 compared to the P group; ** statistical significance at level *p* < 0.01 compared to the P group; # statistical significance at level *p* < 0.05 compared to the P+E1 group; ## statistical significance at level *p* < 0.01 compared to the P+E1 group; and $ statistical significance at level *p* < 0.05 compared to the P+E2 group.

The antioxidant defense system showed alterations following the treatments applied. The superoxide dismutase (SOD) activity was significantly higher in the groups treated with the highest dose of EMPA and MET compared to the P group, as well as in the group treated with the lowest dose of EMPA (*p* < 0.05). Catalase (CAT) activity was significantly higher in all of the treatment groups compared to the P group (*p* < 0.05, *p* < 0.01). Glutathione (GSH) concentration was statistically significantly higher in the middle and highest dose groups treated with EMPA and MET compared to the P group (*p* < 0.05 and *p* < 0.01) (Figure 7).

**Figure 8:**
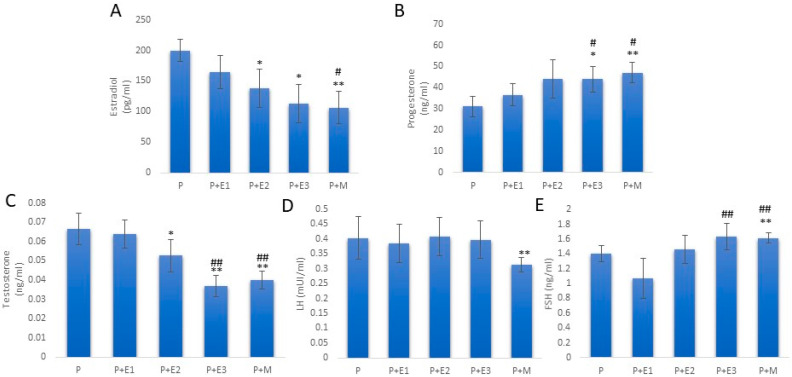
The levels of sex hormones in different groups. (**A**) estradiol levels; (**B**) progesterone levels; (**C**) Testosterone levels; (**D**) FSH levels; and (**E**) LH levels.Bars represent the means ± SEM. * statistical significance at level *p* < 0.05 compared to the P group; ** statistical significance at level *p* < 0.01 compared to the P group; # statistical significance at level *p* < 0.05 compared to the P+E1 group; and ## statistical significance at level *p* < 0.01 compared to the P+E1 group.

The serum estradiol levels were statistically significantly lower in the groups treated with the middle and highest doses of EMPA, as well as after treatment with MET compared to the P group (*p* < 0.05, *p* < 0.01). The serum progesterone levels were statistically significantly higher in the P+E3 and P+M groups compared to the P group (*p* < 0.05 and *p* < 0.01). The groups treated with the middle and highest doses of EMPA (the P+E2 and P+E3 groups), as well as the group treated with MET (P+M group), showed statistically significantly lower serum testosterone levels compared to the P group (*p* < 0.05 and *p* < 0.01). Only MET administration led to a statistically significant decrease in the serum LH and FSH levels compared to the P group (*p* < 0.01). Additionally, the P+E3 and P+M groups exhibited statistically higher serum FSH levels compared to the P+E1 group (*p* < 0.01) (Figure 8).

### 3.3. Impact of EMPA and MET on the PCOS-Related Alteration of Ovarian and Adipose Tissue Morphology

**Figure 9:**
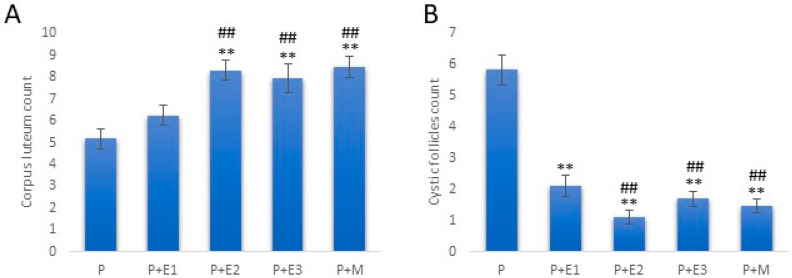
(**A**) the corpus luteum count; (**B**) the cystic follicles count. Bars represent the means ± SEM. ** statistical significance at level *p* < 0.01 compared to the P group; ## statistical significance at level *p* < 0.01 compared to the P+E1 group.

Statistically significant increases in the number of corpora lutea were observed in the P+E2, P+E3, and P+M groups compared to the P group. Additionally, these groups had significantly higher numbers of corpora lutea compared to the P+E1 group. Conversely, the number of cystic follicles was significantly reduced in all treatment groups compared to the P group. Moreover, the P+E2, P+E3, and P+M groups exhibited significantly fewer cystic follicles compared to the P+E1 group (Figure 9).

The number of primary follicles did not show significant differences between groups. Table 2 presents the quantified follicle values from the ovarian sections. All of the doses of EMPA led to a significant increase in the number of secondary follicles compared to the P group, which was similar to the effect of MET. Furthermore, the group receiving the highest dose of EMPA and MET showed significantly more secondary follicles compared to the group receiving the lowest dose of EMPA (*p* < 0.01). The number of tertiary follicles was significantly higher in the groups receiving the highest dose of EMPA and MET compared to the P group.

Administration of the mean and highest doses of EMPA resulted in a significant reduction in the number of atretic follicles compared to the P group, which was similar to the effect of MET. Additionally, the group treated with the highest dose of EMPA and the group treated with MET had significantly fewer atretic follicles compared to the group treated with the lowest dose of EMPA (*p* < 0.01).

**Figure 10:**
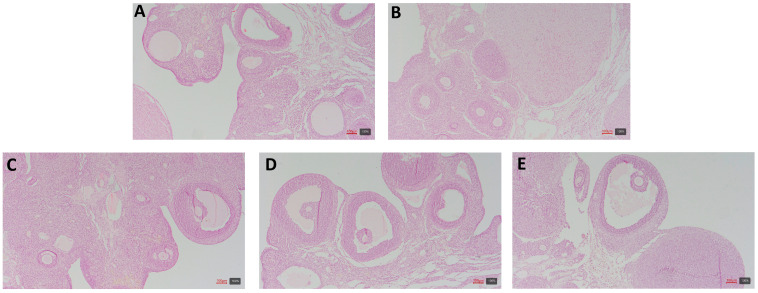
Representative views of the ovaries of the examined groups. (**A**) the P group; (**B**) the P+E1 group; (**C**) the P+E2 group; (**D**) the P+E3 group; and (**E**) the P+M group.

The animals in the P group exhibited altered ovarian tissue structures that were characterized by numerous cystic dilated follicles. Many follicles lacked registered oocytes, and the number of corpora lutea, which is indicative of the previous ovulation, was lower compared to the other groups. The cystic follicles displayed a thinned wall with fewer granulosa cells (3–4 layers). In contrast, treatment with varying doses of EMPA and MET resulted in follicles at different stages of development, with a higher presence of corpora lutea being noted. Developing follicles showed a regular arrangement of granulosa cells in multiple layers (approximately 10 layers), forming thicker follicle walls (Figure 10).

**Figure 11:**
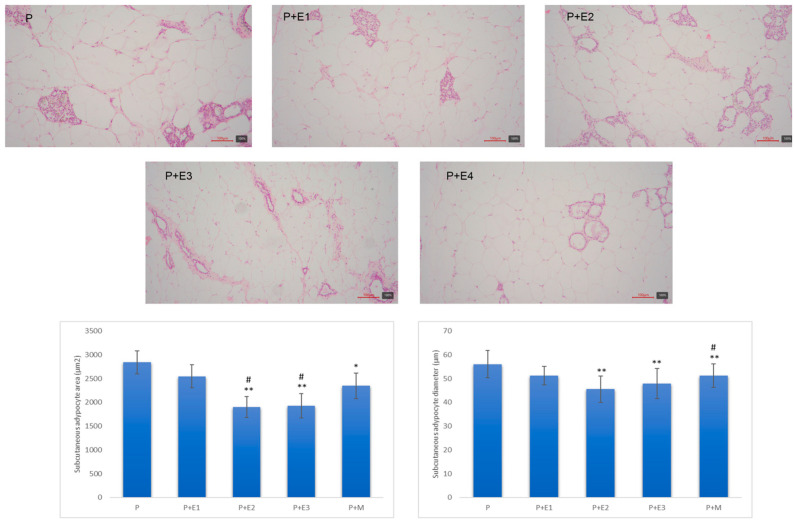
The surfaces and diameters of the subcutaneous fat tissue. Bars represent the mean values ± SEM. * statistically significant difference compared to the P group *p* < 0.05; ** statistically significant difference compared to the P group *p* < 0.01; and # statistically significant difference when comparing the P+E2 and P+E3 groups with the P+E1 group *p* < 0.05.

As shown in Figure 11, after the administration of EMPA in the middle and highest doses, as well as after the administration of MET, there was a statistically significant reduction in the area of subcutaneous fat tissue compared to the P group. Also, the P+E2 and P+E3 groups showed a statistically significant difference compared to the P+E1 group. The diameter of subcutaneous adipose tissue was not statistically different from the P group; rather, it was only found in the P+E1 group. Also, the P+M group was statistically significantly different from the P+E1 group, which is shown in Figure 11. The upper part of the figure shows the representative sections of the subcutaneous fat tissue of the animals.

**Figure 12:**
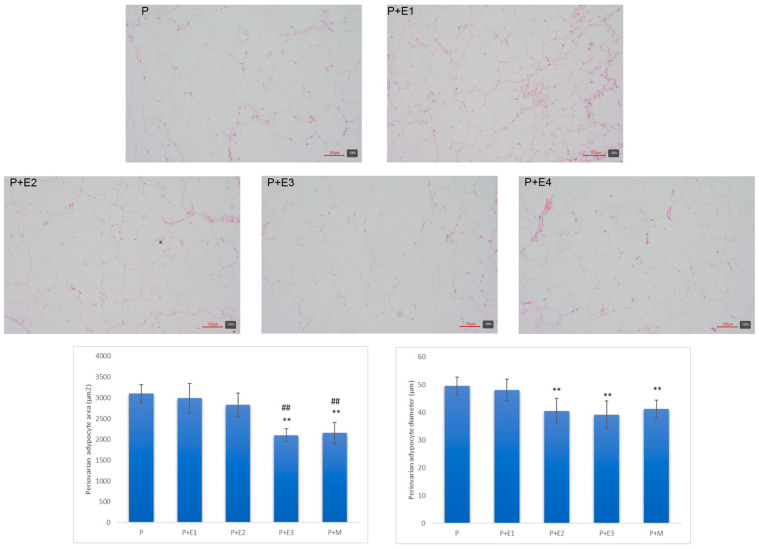
The surface area and diameter of the adipocytes of periovarian fat tissue. Bars represent the mean values ± SEM. ** statistically significant difference compared to the P group *p* < 0.01; and ## statistically significant difference when comparing the P+E3 and P+M groups with the P+E1 group *p* < 0.01.

As shown in Figure 12, after the administration of EMPA at the highest dose, as well as after the administration of MET, there was a statistically significant reduction in the area of visceral adipose tissue compared to the P group, as well as the P+E1 group. Also, in the groups that received the middle and highest dose of EMPA, as well as metformin, there was a decrease in the diameter of visceral fat compared to the P group. In the upper part of Figure 12, representative sections of the periovarian fat tissue of animals are presented.

## 4. Discussion

Research into the application of new drugs, as well as the application of existing drugs for new indications, are the topics that are constantly in the focus of the scientific public. This is particularly important considering that some conditions and diseases still hide complex pathophysiological mechanisms that are not fully understood. Elucidation of these pathophysiological bases may not only contribute to our understanding of the disease, but they may also open the door to the development of new therapeutic approaches that may be meaningful to patients. Considering the existence of interesting data on the application of EMPA in PCOS patients in the recent literature originating from clinical studies, and from the limited data related to the application of EMPA in animal experimental PCOS, the guiding idea for this research was to complete the picture of the effects of the application of different doses EMPA in an animal experimental model. The results of our study show that the administration of EMPA, similar to metformin treatment, exerts significant beneficial effects on both the reproductive and metabolic disorders in a PCOS rat model, which is already known to exert beneficial effects in the regulation of various pathophysiological features related to PCOS. Our results showed that EMPA at the highest dose had a superior effect on the body weight compared to the lower doses of EMPA and MET administration. We recorded a lower level of body mass. However, the amount of adipose tissue was also, among other things, accompanied by a regulation of the glucose level and hyperandrogenemia in the PCOS model. Administration of a medium dose of EMPA and administration of metformin led to a moderate decrease in the final body weight, while administration of a high dose of EMPA caused a more pronounced loss of body weight. The effects of MET on the body weight regulation in PCOS have been previously described [46]. Our obtained results regarding EMPA treatment are consistent with previous studies regarding the effects of EMPA on the anthropometric parameters in patients [47]. Furthermore, the literature data show superior effects of EMPA in PCOS patients compared to MET in regulating body weight. Our results showed that EMPA at the highest dose has a superior effect on body weight compared to lower doses of EMPA and metformin administration. However, effects on hormonal and metabolic parameters were not realized in the mentioned study [27]. This result is not in agreement with our study conducted on the PCOS model in rats when it comes to the regulation of hormonal status, as will be explained in more detail below. The use of EMPA can improve the somatometric, metabolic, and hormonal outcomes of PCOS. In our model of induced PCOS on rats, we recorded a lower level of body mass and the estrus cycle, but also the amount of adipose tissue, which, among other things, was accompanied by the regulation of glucose levels and hyperandrogenemia in the PCOS rat model.

Although it is considered that the application of a PCOS induction protocol using a single dose of estradiol valerate is not an adequate way through which to establish the metabolic status characteristic of PCOS patients [34,48,49], an elevated blood glucose level and impaired glucose regulation with insulin resistance has been proven after such a protocol [50,51]. In addition, Daneasa et al. found elevated fasting blood glucose levels in the PCOS group, but no difference was found between the PCOS and controls after OGTT was applied [52]. In our study, a PCOS induction protocol using EVs with a high-fat diet regimen resulted in higher blood glucose levels in PCOS rats compared to the control group after 30 and 60 min of OGTT [22]. Both in the 30th minute and in the 60th and 120th minutes during the performance of the OGTT, the glycemic values were lower in all of the groups treated with EMPA, as well as in the group treated with metformin, when compared to the PCOS group. The lowest glycemic values during the OGTT in all minutes were in the group with the highest applied dose of EMPA. It was interesting that none of the groups treated with EMPA or metformin had glycemic levels below the reference range. In addition, the analysis of the area under the glycemic curve showed a higher value in the PCOS group compared to all other groups. This result should be kept in mind when discussing the improvement of glycoregulation because disturbances in glucose and insulin secretion are very common comorbidities of PCOS. The primary effect of EMPA is achieved by lowering the level of glucose in the blood [53] in an insulin-independent way, thereby acting on the proximal tubules of the kidneys and preventing glucose reabsorption. Meanwhile, metformin, which is still considered the main agent in the treatment of PCOS, reduces the intestinal absorption of glucose. Numerous studies have shown that metformin improves the body composition and insulin levels in non-obese women with PCOS, but it does not affect BMI or basal glucose [54], and the use of EMPA for these purposes can be considered valuable. EMPA is thought to lower body weight (by 1.8–2.7 kg) with little likelihood of causing hypoglycemia [55]. Unlike other antidiabetic drugs, the lowering of plasma glucose with EMPA is independent of pancreatic beta cell function and insulin sensitivity. This insulin-independent lowering of plasma glucose reduces insulin requirements and causes an increase in the glucagon-to-insulin ratio, shifting the metabolism into a catabolic state [56]. PCOS is characterized by tissue-selective insulin resistance [57]. Selective insulin resistance prevents glucose metabolism in muscles and fat tissue, resulting in hyperinsulinism that worsens the condition of hyperandrogenemia [57]. Although, according to the valid Rotterdam criteria, hyperinsulinemia is not a diagnostic criterion for PCOS, the etiopathogenetic mechanism of this disorder is important given that there is a close connection between the hyperandrogenism and hyperinsulinemia in patients with insulin resistance, PCOS, and DM2 [58]. Hyperinsulinemia in PCOS increases the secretion of LH, and through the indirect action of LH, therefore, enhances the secretion of androgens in the ovaries. Also, the success of increased insulin secretion leads to a reduced production of SHBG in the liver, which increases the availability of the free testosterone in circulation.

In terms of blood pressure, all of the treated groups exhibited decreased systolic blood pressure, while the diastolic blood pressure only decreased in the middle-dose EMPA group. The heart rate decreased in the low- and middle-dose EMPA groups, and the pulse pressure remained similar across groups. However, the mean arterial pressure (MAP) significantly decreased only in the middle-dose EMPA group. These cardiovascular effects suggest a potential role for EMPA in mitigating the hypertension associated with PCOS, contributing to the overall cardiovascular benefits observed in these patients. The observed decrease in the systolic blood pressure with EMPA corresponds with the studies indicating similar cardiovascular benefits in diabetic and non-diabetic conditions [59]. EMPA at a dose of 30 mg/kg per day over 6 weeks improves the systolic blood pressure and endothelial dysfunction in rats with metabolic syndrome [60]. Multiple studies have indicated a higher incidence of hypertension among women with PCOS compared to the general population [61]. Moreover, in studies examining the blood pressure in PCOS, there has been registered beneficial effect of EMPA. In particular, Pruett et al. demonstrated that treatment with EMPA dissolved in drinking water at a dose of 10 mg per kilogram of BW during the last three weeks of dihidrotestosterone administration to induce PCOS affects the blood pressure. Their study revealed that blocking SGLT2 with EMPA leads to a decrease in fat mass but also blood pressure. However, it does not improve insulin resistance in an animal model of PCOS. The observed reduction in blood pressure with EMPA treatment in PCOS rats were attributed by the authors to an improvement in the intrarenal ACE expression and activity induced by androgens [29]. Moreover, metformin treatment reduced the systolic blood pressure, similar to the EMPA treatments, in our study. The cause of hypertension in women with PCOS remains unclear as there is no definitive evidence directly linking it to androgens or hyperinsulinemia. Metformin decreased systolic blood pressure in our study, which is in line with previous reports [62]. However, MAP was decreased only by the middle-dose EMPA in our study, while metformin did not significantly alter this parameter. However, other studies have shown that metformin treatment is able to reduce MAP in insulin-resistant rats, which could be linked to a direct impact on the endothelium and nitric oxide–dependent relaxation; however, the exact underlying mechanism remains uncertain [63]. Alternatively, the reduction in MAP by metformin may be associated with its insulin-sensitizing properties, while suggesting that metformin improves vascular function through a direct mechanism rather than by improving metabolic abnormalities. However, in a study on systolic blood pressure, the EMPA-treated group did not reach statistical significance compared to the non- treated PCOS group. Heart rate was decreased in the low-dose and middle-dose EMPA-treated rats compared to PCOS group without treatment. Regarding the mentioned EMPA treatment, our results differ from other studies, which have shown no difference in heart rate and heart rate variability after EMPA treatment [64,65]. However, the high-dose EMPA and metformin treatment used in our study did not alter the heart rate, while these doses were able to reduce the systolic blood pressure, which is in accordance with the previously mentioned studies.

Biochemical hyperandrogenism, such as increased levels of T and/or DHEAS, are one of the diagnostic criteria of PCOS [9]. A disturbed hypothalamic–pituitary–ovarian axis is a feature of RCOS that disrupts the hormonal secretion in physiological rhythms, primarily causing an increased secretion of LH and testosterone [66]. In our PCOS estradiol valerate induction model, we added a high-fat diet regimen because a high-fat diet is believed to act on reproductive neurons by altering their secretion patterns (since the pulsatile release of GnRH and LH is necessary for further follicular maturation) [67]. EMPA in the middle and highest doses, as well as metformin, lowered serum testosterone levels. However, it was only the lowest administered dose of EMPA that did not also lead to a decrease in serum estradiol. The highest applied dose of EMPA, as well as the use of metformin, led to an increase in the level of progesterone in the serum, which can lead us to the conclusion that, due to the use of EMPA in the highest dose that we used, as well as the use of metformin, the ovulation and the formation of the corpus luteum occurred, which it then consequently produced. The use of EMPA and metformin, given that we came to the result after treatment with EMPA and metformin testosterone secretion decreased and estradiol and progesterone secretion increased, improved the dynamics of the folliculogenesis in the ovaries and reduced the secretion of the androgens. The use of EMPA did not lower the LH secretion in our experimental model, but a previous study also showed that SGLT2i lowers androgen secretion not by acting on LH secretion, but by improving the activity of pancreatic beta cells with a reduced need for insulin and with a reduction in hyperinsulinemia, which are the main factors contributing to the increase in levels of testosterone in PCOS [68]. The increase in FSH levels was caused by the highest applied dose of EMPA, as well as metformin administration. EMPA had a beneficial effect on the hormonal status because it improved the endocrine function and insulin resistance, thereby significantly reducing the testosterone levels and inducing improvements in the estradiol, progesterone, LH, and FSH levels. 

Oxidative stress (OS) represents the excessive production of free oxygen radicals (reactive oxygen species—ROS). The excessive production of ROS causes disturbances between oxidative stress, disturbances of homeostasis between the oxidation and antioxidant defense systems, and it also leads to the loss of the organism’s ability to fight against ROS, thus creating cellular damage. The cell damage caused by ROS is at the level of macromolecules, lipids, proteins, and nucleic acids [69]. Conditions that often coexist with PCOS such as centripetal obesity, dyslipidemia, and insulin resistance also promote the formation of oxygen species and the occurrence of oxidative stress. EMPA has been shown to alleviate oxidative stress in mitochondria [70], and it has been shown that mitochondria are essential in the regulation of oxidative stress. In addition, it has also been shown that women with PCOS have mitochondrial dysfunction with the increased production of oxygen free radicals and reduced ATP production [71]. In our study, the level of TBARS was significantly reduced after the administration of the middle and highest doses of EMPA, as well as after administration of metformin. Application of metformin, as well as EMPA at the highest dose, led to the greatest lowering of TVARS in all of the investigated groups. The obtained results of our study are in accordance with the results of similar studies [72,73]. The concentration of O_2_^−^ did not change significantly in all of the treated groups compared to the untreated group. At all applied doses of EMPA, as well as with metformin administration, there was a decrease in the value of H_2_O_2_ in the plasma, and the alterations in the values between the groups were similar. It is interesting that only the lowest dose of EMPA led to a decrease in plasma NO_2_^−^ values. Only the highest administered dose of EMPA, as well as the administration of metformin, led to an increase in SOD activity and an improvement in the antioxidant effect in the blood, but all of the doses of EMPA, as well as metformin, were able to increase SAT activity. The level of GSH was decreased in the P group, which is in accordance with the already published animal studies when taking into account the fact that the predominance of prooxidants exists in this syndrome [74]. Only the lowest administered dose of EMPA failed to increase the GSH activity, while the increased GSH protection in ovarian tissue was noted following administration of the highest dose of EMPA and metformin. From all of the above, we can conclude that, in order to improve the antioxidant defense system, EMPA, as well as metformin, can be used in the highest applied dose. And since EMPA improves mitochondrial function, the normal functioning of which is disturbed in PCOS, its application in the treatment of these disorders may be promising.

LH secretion regulates androgen synthesis, enables initiation of meiotic division in oocytes, induces ovulation, and develops the corpus luteum [75]. FSH enables granulosa proliferation and induces estrogen synthesis, while it also acts on follicle development from the primary to the preantral follicle stage [75]. It was observed that estradiol valerate prevents the normal maturation of follicles and increases the number of cystic follicles, and it also decreases the number of corpora lutea, which indicates that ovulation does not occur as in physiological conditions. The results of our study show that the use of EMPA, as well as the use of MET, lead to a decrease in the number of cystic follicles. In addition, it also leads to an increase in the number of corpora lutea in the middle and highest doses of EMPA, as well as due to metformin therapy, which implies that, due to EMPA therapy, as well as metformin, it increases the rates of ovulatory cycles. According to the results of our research and studies whose research focus was similar to ours, it showed a reduced number of cystic follicles with a thickening of the granulosa cell layer after the application of EMPA and metformin [68]. The same study [68] pointed out that, in their PCOS model, after EMPA and metformin therapy, the number of de Graaffian follicles increased, and the pre-antral, atretic follicles with a reduced number of antral follicles and corpora lutea were dominantly present before therapy [76,77] The results of our study showed that all of the applied doses of EMPA, similar to metformin, led to a significant increase in the number of secondary follicles compared to the untreated group. The number of tertiary follicles was significantly higher in the groups treated with the highest dose of EMPA, as well as metformin. The mean and highest administered dose of EMPA, similar to metformin, led to a significant reduction in the number of atretic follicles compared to the P group. Research on a PCOS model in mice also revealed that, due to the administration of metformin, there was a decrease in the number of primary, atretic, and cystic follicles, as well as an increase in the number of corpora lutea and graph follicles [78].

In our study, a positive effect of EMPA on reducing the surface area and diameter of visceral adipose tissue was shown. Application of EMPA in the highest dose, as well as application of metformin, resulted in a statistically significant reduction in the surface area of visceral adipose tissue adipocytes and a decrease in the diameter of visceral adipose tissue adipocytes. Also, we proved that EMPA in the middle and highest doses that we applied, as well as metformin, led to a statistically significant reduction in the area and diameter of subcutaneous adipose tissue adipocytes, and this effect was similar to the effect of metformin. Similar results, in terms of adipocyte surface area reduction, were obtained by researchers who analyzed the effect of SGLT2i on the mitochondrial function and oxidative stress in adipose tissue [29]. In addition, in a study in prediabetic rats, EMPA treatment significantly increased the percentage of smaller-diameter adipocytes, while decreasing the percentage of larger-diameter adipocytes in visceral (epididymal) adipose tissue, indicating a beneficial effect of EMPA in reducing visceral adipocyte hypertrophy [79]. This effect was associated with a reduction in oxidative stress in visceral but not in subcutaneous adipose tissue. Similarly, our results confirmed that the middle and highest applied doses of EMPA reduce the concentrations of pro-oxidative parameters to the greatest extent, with a consequent increase in the markers of antioxidant protection in the blood and in the ovary. In addition, the decrease in testosterone concentrations in these animals is consistent with a decrease in adipocyte size. There is evidence that signaling through the androgen receptor at the level of adipose tissue is crucial for the development of metabolic aspects [80]. Interactions at the level of adipose tissue and reproductive functions are explained by signaling through adipokines. Elevated concentrations of adipose tissue-derived adipokines in PCOS may stimulate kisspeptin neurons in the hypothalamus, which further leads to an increased frequency of gonadotropin-releasing hormone release. This stimulation is reflected in an increase in androgen secretion, which is a key link in the development of PCOS symptoms. Studies on rats that were exposed to a diet with an increased fat content showed that such an intervention can affect the regularity of the ovarian cycle, precisely by increasing the frequency of pulsatility of luteinizing hormone, but also by increasing the expression of kisspeptin [81]. In this way, hyperinsulinemia and ovarian hyperandrogenism can be associated with infertility and obesity in PCOS [82].

## 5. Conclusions

**Scheme 1:**
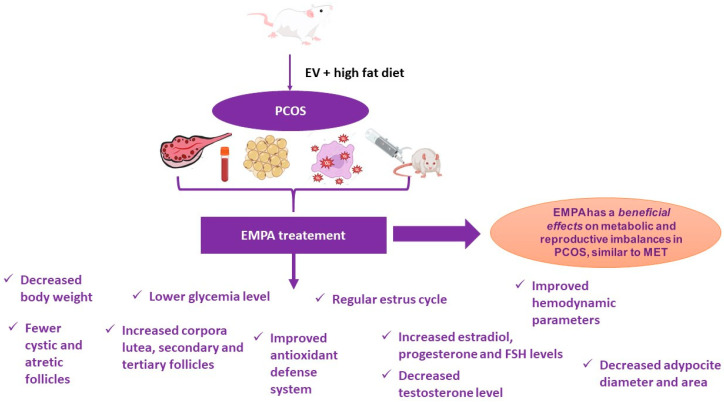
Summary scheme of the effects of EMPA in a PCOS rat model. PCOS—polycystic ovary syndrome; EMPA—empagliflozin; and MET—metformin.

This study demonstrated that EMPA), comparable to metformin, exerts significant beneficial effects on both the reproductive and metabolic disorders in a rat model of PCOS (Scheme 1). EMPA, particularly at higher doses, effectively addresses the key features of PCOS, including weight management, glucose regulation, hormonal imbalances, and ovarian and adipose tissue morphology. EMPA also shows positive effects on cardiovascular parameters and oxidative stress markers. These findings collectively suggest that EMPA could be a promising therapeutic option for PCOS, addressing both reproductive and metabolic aspects of the syndrome. The dose-dependent effects observed highlight the importance of optimizing dosage in potential clinical applications. Future research should focus on long-term effects, potential side effects, and clinical trials to validate these findings in human subjects. Despite the valuable insights provided, this study has some limitations. The use of a rat model, while necessary for mechanistic investigations, may not fully replicate the complex pathophysiology of human PCOS, potentially limiting the direct translation of results to clinical practice. Additionally, the relatively short treatment duration of 28 days may not capture the long-term effects or potential side effects of SGLT2i use in PCOS. Future studies with longer treatment periods and investigations in human subjects would be beneficial to further validate these findings and assess their clinical applicability.

## Figures and Tables

**Table 1:** Results of the oral glucose tolerance test in rats. The glucose concentration in the plasma at 0, 30, 60, 120, and 180 min. Data are presented as the mean ± SEM. ** statistically significant difference compared to the P group (*p* < 0.01), # statistically significant difference compared to the P group (*p* < 0.05).

Glucose (mmol/L)	0′	30′	60′	120′
P	7.63 ± 1.03	10.29 ± 0.88	8.356 ± 0.77	7.34 ± 0.58
P+E1	5.05 ± 1.10 **	8.30 ± 1.92 **,#	7.08 ± 1.65 **,#	5.53 ± 1.29 **
P+E2	4.78 ± 0.32 **	7.68 ± 0.55 **,#	6.36 ± 0.22 **	5.10 ± 0.34 **
P+E3	5.22 ± 0.25 **	7.02 ± 0.50 **	6.02 ± 0.32 **	5.26 ± 0.31 **
P+M	5.26 ± 1.31 **	7.93 ± 1.91 **,#	6.57 ± 1.59 **	5.63 ± 1.34 **

**Table 2:** The number of different types of follicles in the cross-sections of the ovarian tissue. The values are presented as the means ± SEM. * statistical significance at level *p* < 0.05 compared to the P group; ** statistical significance at level *p* < 0.01 compared to the P group; ## statistical significance at level *p* < 0.01 compared to the P+E1 group.

Follicle Types	P	P+E1	P+E2	P+E3	P+M
Primary	10.67 ± 5.13	12.33 ± 3.21	15.33 ± 1.15	16 ± 2.64	21 ± 5
Growing	7.33 ± 0.57	12.67 ± 2.08 *	16 ± 1 **	20.67 ± 3.79 **,##	21.67 ± 3.05 **,##
Graafian	3 ± 1.00	3.67 ± 1.53	5 ± 1 *	5.33 ± 1.15 *	5.67 ± 2.08 **
Аtretic	13.33 ± 1.53	11 ± 1	7.67 ± 0.58 *	5.67 ± 2.08 **,##	6.67 ± 2.3 **,##

## Data Availability

The raw data supporting the conclusions of this article will be made available by the authors on request.
